# Infective endocarditis of quadricuspid aortic valve

**DOI:** 10.1186/s13019-023-02164-x

**Published:** 2023-02-07

**Authors:** Ariana M. Goodman, Hossein Amirjamshidi, Daniel R. Ziazadeh, Andrew S. Jones, Kazuhiro Hisamoto

**Affiliations:** 1 Department of Surgery Division of Cardiac Surgery, University of Rochester Medical Center, Box: Surg 601 Elmwood Ave, Rochester, NY 14642 USA; 2 School of Medicine and Dentistry, University of Rochester, Rochester, NY USA

**Keywords:** Endocarditis, Bacterial endocarditis, Aortic valve, Congenital aortic valve, Infective endocarditis, Cardiac surgery, Surgical aortic valve replacement

## Abstract

**Background:**

Infective endocarditis of the aortic valve is a relatively common disease presentation, with surgical intervention a mainstay of treatment in severe cases. Quadricuspid aortic valves are a rare spontaneous developmental anomaly that are more likely to be asymptomatic, and less likely to require a full valve replacement than their hypocuspid counterparts. However, there is very little literature addressing infective endocarditis of this valve variant.

**Case presentation:**

This case report presents a case of infective endocarditis of a quadricuspid aortic valve that required replacement with a surgical bioprosthetic valve. The patient is a 30 year old male with a history of polysubstance use, upper extremity aneurysm, and prior tricuspid valve endocarditis. Surgical aortic valve replacement was performed with a 25 mm tissue valve via median sternotomy.

**Conclusions:**

The patient made a full recovery after surgical aortic valve replacement and a course of antibiotics and was discharged home without any complications. This supports that surgical aortic valve replacement is feasible and safe in patients with polycuspid aortic valve endocarditis.

## Background

Infective endocarditis occurs due to a variety of factors including the type of organism implicated, the structure of the heart valve that it adheres to, and the ability for the organism to maintain that adherence. People who inject drugs (PWID) are at risk for infective endocarditis due to the increased likelihood of bacterial and fungal introduction into the blood stream. There has been an overall increase in hospital admissions due to infective endocarditis over the last 30 years. Between 2000 and 2013, hospitalizations for infective endocarditis rose 38%, but those for endocarditis secondary to IV drug use increased over 200% [1].

The most commonly affected heart valve in IV drug users with endocarditis is the tricuspid valve, making up between 58 and 77% of cases, while surgical intervention most often occurs on the aortic valve [1]. Our patient developed enterococcus faecalis endocarditis of his quadricuspid aortic valve, which is the third most frequent organism implicated in infective endocarditis [2]. Unlike in pathologies of bicuspid aortic valve or even endocarditis of a tricuspid aortic valve, there are no guidelines to aid in surgical decision making of quadricuspid aortic valves.

## Case presentation

Patient is a 30-year-old male with history of polysubstance use, current tobacco use, upper extremity aneurysm with history of right brachial artery bypass with chronic lymphedema, tricuspid valve endocarditis treated with antibiotics only three years prior to presentation who initially presented with left upper quadrant pain. He was found to be bacteremic with blood cultures growing Enterococcus faecalis. He was initially started on Vancomycin and then transitioned to Ampicillin and Ceftriaxone. Imaging revealed a splenic embolic lesion on CT scan thought to be secondary to abscess or infarct. A Trans Esophageal Echocardiogram (TEE) was obtained which revealed a quadricuspid aortic valve with multiple vegetations on the ventricular aspect of all four leaflets, the largest of which measured 1.3  × 1.4 cm. He was also found to have moderate to severe aortic regurgitation with diastolic flow reversal in the proximal descending thoracic aorta.

His clinical course was complicated by new onset numbness, weakness, and tingling of his extremities as well as slurred speech. MRI Head was consistent with a moderately sized subcortical ischemic infarct. The patient had improvement in functionality following the. Neurosurgery and neurology were both consulted. While they had recommended holidng off on systemic anticoagulation due to the risk of hemorrhagic transformation, they did not feel that a thrombectomy was required. A digital subtraction angiography (DSA) was performed to look for mycotic aneurysms, which was negative, although it did show a distal right V3 segment saccular aneurysm measuring 4.72 × 3.16 × 3.63 mm with a 3.1 mm neck. The patient did not have permanent deficits from the stroke.

Despite the lack of heart failure symptoms, it was determined that surgical intervention was required due to moderate to severe aortic regurgitation, the size of the vegetations and evidence of septic emboli. Given the risk of hemorrhagic transformation with cardiopulmonary bypass and heparin administration, surgical intervention of his aortic valve was delayed for two weeks following the patient’s stroke per the recommendation of Vascular Neurology. Repeat CT scan on the day prior to surgery showed an evolving subacute infarct in the M2 distribution with no evidence of hemorrhagic transformation.

A surgical aortic valve replacement was performed via median sternotomy. An Inspiris Resilia aortic valve® (INSPIRIS) (Edwards Lifesciences LLC, Irvine, USA) was used via a supra-annular technique due to patient preference and concern for Warfarin compliance The surgery was uneventful. Cardiopulmonary bypass time was 88 min and cross clamp time was 60 min. Despite a previous history of tricuspid valve endocarditis, the patient did not have any tricuspid insufficiency or evidence of vegetation on TEE, and thus we did not intervene on the tricuspid valve. The patient was extubated on post-operative day zero and transferred out of the ICU on post-operative day one. Soon after, he was transferred to the medicine-psychiatric inpatient unit that specializes in addiction medicine, allowing the patient to complete a full course of antibiotics while starting his recovery process. He completed 5 weeks of IV antibiotics as an inpatient and was given a single dose of Dalbavancin to cover an additional 7–10 days.

## Discussion

While we operate on many patients with infective endocarditis at our institution, what separates this patient from others with IE is the presence of a quadricuspid aortic valve. His IV drug use as well as his valvular abnormality increases his risk factors for development of IE. There is little research on quadricuspid valve, and even less on IE in these patients.

The incidence of quadricuspid aortic valve is quite low, ranging from 0.003% in some studies [3] to 0.043% in others [3, 4]. The embryologic mechanism of quadricuspid valve formation is still unknown, but it’s thought to be secondary to abnormal septation of the arterial trunk [4]. Aberrant cusp formation may represent fusion of the aorticopulmonary septum or abnormal mesenchymal proliferation in the truncus arteriosus [5]. While it is normally an isolated anomaly, additional congenital cardiac anomalies can be present in up to 32% of patients [4].

**Fig. 1:**
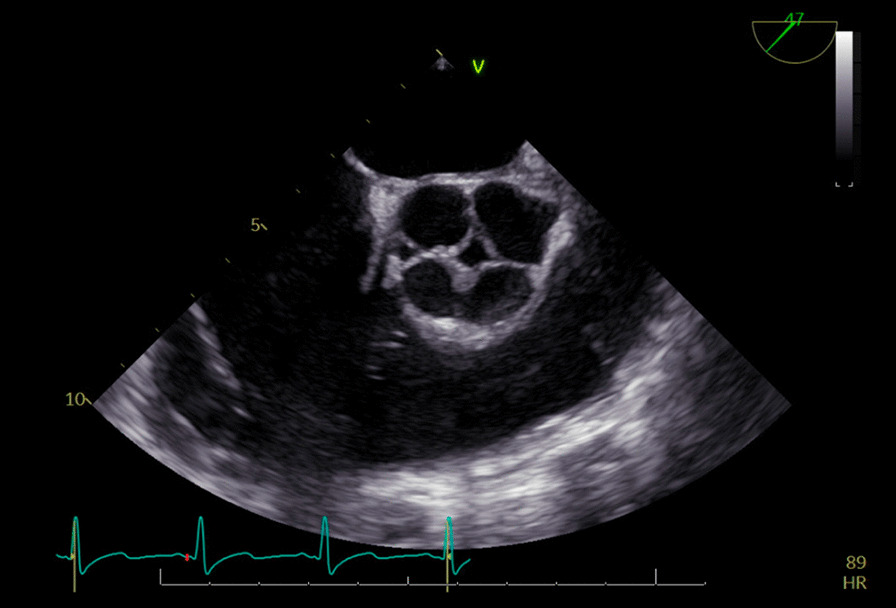
Quadricuspid aortic valve as seen on ECHO

**Fig. 2:**
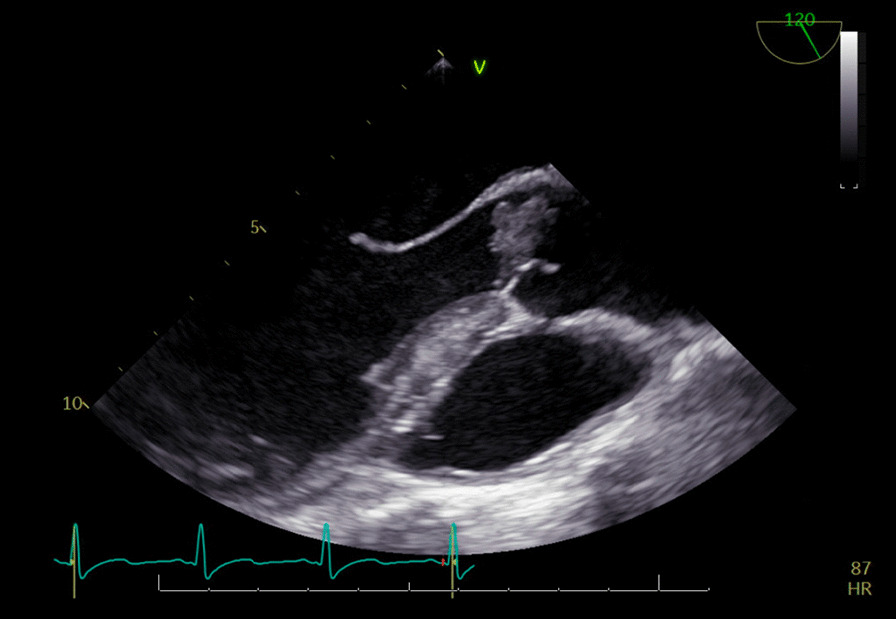
TEE, aortic valve vegetation. There was no evidence of aortic aneurysm. The size of the sinuses of Valsalva, ascending aorta, aortic arch and descending aorta are normal. Aortic Diameters: sinus = 3.2 cm, tubular = 3.2 cm

**Fig. 3:**
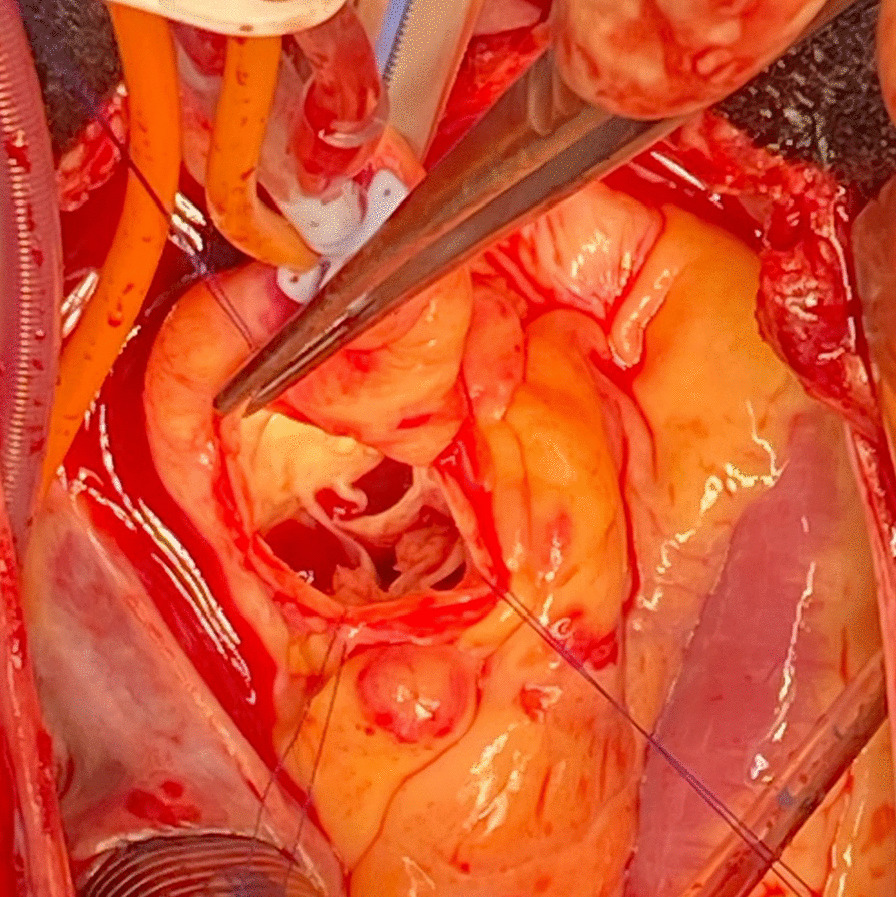
Intraoperative view of quadricuspid aortic valve, in-situ

**Fig. 4:**
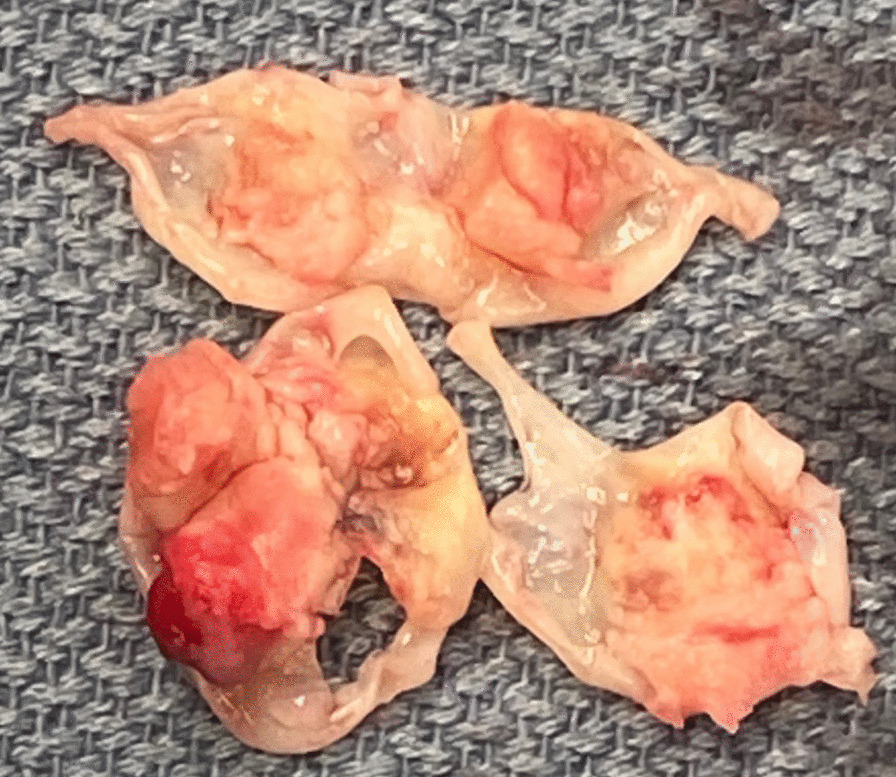
Aortic valve leaflets after removal

It was thought that a small supernumerary cusp can be a predictive risk factor of infective endocarditis, as unequal cusps lead to uneven distribution of stress and progressive aortic regurgitation and deterioration over time [5]. However, there have also been case report studies of patients with equal sized quadricuspid cusps who have still developed IE, and even perforation [6]. This patient was found to have four equal sized cusps (Figs. 1, 2, 3, 4) and thus the valve falls into the category of a type A valve, as outlined by Hurwitz and Robert [7] in 1973 who created a classification scheme for quadricuspid valves based on the relative size of the supranumery cusp [5]. TEE prior to surgery also showed multiple extensive tissue-density masses on the inflow side of the aortic leaflet cusps and moderate aortic regurgitation (Fig. 2) which fits with the data that shows increased prevalence of aortic regurgitation rather than stenosis in patients with quadricuspid aortic valves. [8]

Early surgical intervention has been considered in many cases with persistent bacteremia, ongoing embolic phenomena and large vegetation. The optimal timing of cardiac surgery following an ischemic stroke is unknown, stating that surgery should be delayed between 2 and 4 weeks. However, based on review of current literature, it is suggested that, that cardiac surgery is beneficial when indicated and has a significant reduction in mortality compared to conservative treatment [9]. Tricuspidization and annular banding technique has been reported as the preferred surgical technique in many case reports for quadricuspid aortic valve repair. However, given the extent of involvement and large vegetations we elected to proceed with aortic valve replacement.

## Conclusion

There are no set guidelines or management recommendations regarding surgical indication, surgical procedures, or antibiotic prophylaxis against IE in patients with quadricuspid aortic valves. Current management decisions are made clinically by the physician and are similar to the BAV guidelines and are based on the size of the vegetation and clinical picture.

## Data Availability

All data generated or analyzed during this study are included in this published article [and its supplementary information files].
